# Alternative polyadenylation-related genetic variants contribute to bladder cancer risk

**DOI:** 10.7555/JBR.37.20230063

**Published:** 2023-11-15

**Authors:** Ting Liu, Jingjing Gu, Chuning Li, Mengfan Guo, Lin Yuan, Qiang Lv, Chao Qin, Mulong Du, Haiyan Chu, Hanting Liu, Zhengdong Zhang

**Affiliations:** 1 Department of Environmental Genomics, Jiangsu Key Laboratory of Cancer Biomarkers, Prevention and Treatment, Collaborative Innovation Center for Cancer Personalized Medicine, School of Public Health, Nanjing Medical University, Nanjing, Jiangsu 211166, China; 2 Department of Genetic Toxicology, the Key Laboratory of Modern Toxicology of Ministry of Education, Center for Global Health, School of Public Health, Nanjing Medical University, Nanjing, Jiangsu 211166, China; 3 Department of Urology, Jiangsu Province Hospital of Traditional Chinese Medicine, Nanjing, Jiangsu 210029, China; 4 Department of Urology, the First Affiliated Hospital of Nanjing Medical University, Nanjing, Jiangsu 210029, China

**Keywords:** alternative polyadenylation, genetic variant, bladder cancer, *PRR13*, apaQTL

## Abstract

Aberrant alternative polyadenylation (APA) events play an important role in cancers, but little is known about whether APA-related genetic variants contribute to the susceptibility to bladder cancer. Previous genome-wide association study performed APA quantitative trait loci (apaQTL) analyses in bladder cancer, and identified 17 955 single nucleotide polymorphisms (SNPs). We found that gene symbols of APA affected by apaQTL-associated SNPs were closely correlated with cancer signaling pathways, high mutational burden, and immune infiltration. Association analysis showed that apaQTL-associated SNPs rs34402449 C>A, rs2683524 C>T, and rs11540872 C>G were significantly associated with susceptibility to bladder cancer (rs34402449: OR = 1.355, 95% confidence interval [CI]: 1.159–1.583, *P* = 1.33 × 10 ^−4^; rs2683524: OR = 1.378, 95% CI: 1.164–1.632, *P* = 2.03 × 10 ^−4^; rs11540872: OR = 1.472, 95% CI: 1.193–1.815, *P* = 3.06 × 10 ^−4^). Cumulative effect analysis showed that the number of risk genotypes and smoking status were significantly associated with an increased risk of bladder cancer ( *P*_trend_ = 2.87 × 10 ^−12^). We found that *PRR13*, being demonstrated the most significant effect on cell proliferation in bladder cancer cell lines, was more highly expressed in bladder cancer tissues than in adjacent normal tissues. Moreover, the rs2683524 T allele was correlated with shorter 3′ untranslated regions of *PRR13* and increased *PRR13* expression levels. Collectively, our findings have provided informative apaQTL resources and insights into the regulatory mechanisms linking apaQTL-associated variants to bladder cancer risk.

## Introduction

Bladder cancer ranks the tenth most common cancer, with approximately 573000 new cases and 213000 deaths in 2020 worldwide ^[ 1] ^. In recent years, the incidence and mortality rates of bladder cancer in China have risen, ranking the first among male urological malignancies ^[ 2] ^. Epidemiological studies have demonstrated that bladder cancer prevalence is nearly fourfold higher in men than in women ^[ 1] ^. The development of bladder cancer results from genetic and environmental factors, among which occupational exposure and smoking are the main risk factors ^[ 3] ^. Emerging evidence has suggested that single nucleotide polymorphisms (SNPs) are the cause of genetic susceptibility in individuals ^[ 4] ^. Genome-wide association studies (GWASs) aimed at identifying genetic variants involved in various diseases, including bladder cancer, have become an efficient tool, but the functional elucidation of these genetic variants has been a major challenge ^[ 5] ^. Previously, our team identified some susceptibility loci with biological functions in bladder cancer by conducting GWASs ^[ 6] ^, such as rs5746136, which affects the level of RNA N ^6^-methyladenosine methylation in *SOD2* and is notably associated with bladder cancer risk ^[ 7] ^.

Genetic information requires a series of steps to transcribe DNA into messenger RNA (mRNA) transcripts ^[ 8] ^. The mRNA precursors are processed into mature mRNAs mainly by three post-transcriptional modifications, including 5′-end capping, 3′-end polyadenylation, and RNA splicing ^[ 9] ^. Processing of the 3′-end of mRNA can facilitate pre-mRNA transport out of the nucleus and impact the stability of mature transcripts and the translation efficiency of mRNA ^[ 10] ^. During maturation of the 3′-end of mRNA, most genes usually have multiple polyadenylation signals (PASs) ^[ 11] ^. Alternative polyadenylation (APA), a common post-transcriptional modification ^[ 12] ^, selects to utilize different PASs and results in generating multiple transcript isoforms of genes with a variable length of 3′ untranslated regions (3′ UTRs) ^[ 13] ^. It may alter important regulatory binding sites and thereby affect the stabilization and translation efficiency of the mRNA ^[ 14] ^. The regulation of APA is currently known to be influenced by several factors, such as PAS sequence, the distance between two PASs, chromatin modifications, the elongation rate of the transcriptase, the strength of protein-RNA interaction, and the concentration of core processing factors ^[ 13] ^. In addition, APA is one of the vital mechanisms to regulate gene expression in eukaryotes with tissue specificity, and previous studies have found that its abnormal regulation could lead to diverse diseases, such as hematological, immunological, and neurological diseases, including cancers ^[ 15] ^.

Traditionally, investigators have used expression quantitative trait loci (eQTL) or alternative splicing quantitative trait loci to explain molecular mechanisms ^[ 16– 17] ^. Studies demonstrated that genetic variants correlated with APA were associated with the development of multiple diseases by altering the transcript length in the 3′ UTR of the gene ^[ 18] ^. The SNP2APA database is a new computational pipeline that systematically performs APA quantitative trait loci (apaQTL) analyses on 32 cancer types by utilizing genotype data from The Cancer Genome Atlas (TCGA) and APA data from the Cancer 3′ UTR Atlas (TC3A) ^[ 19] ^.

In the current study, we obtained the apaQTL-associated SNPs from the SNP2APA database and further carried out a case-control study in a Chinese population to explore the correlations with APA-related genetic variants and bladder cancer risk.

## Materials and methods

### Characterization of apaQTL-SNPs and apaQTL-genes

We downloaded the apaQTL-SNPs for bladder cancer from the SNP2APA database ^[ 19] ^. This database quantified APA events by the Percentage of Distal polyA site Usage Index (PDUI) values, with larger PDUI values indicating that more transcripts took advantage of the distal polyA sites, and the reverse was also true ^[ 20] ^. We used the Variant Effect Predictor ( http://grch37.ensembl.org/info/docs/tools/vep/index.html), a tool to analyze and prioritize genetic variants in the coding and noncoding regions, to annotate apaQTL-SNPs.

By using the SNP2APA database, we obtained the gene symbols of APA (apaQTL-genes) that were affected by apaQTL-SNPs. The pathway enrichment analysis was performed with the Kyoto Encyclopedia of Genes and Genomes (KEGG) pathway. The bubble plot was made by https://www.bioinformatics.com.cn, an online platform for data analysis and visualization. The somatic copy number alterations (SCNAs) were analyzed by a module called GISTIC 2.0 in GenePattern ( https://cloud.genepattern.org/gp/pages/index.jsf). The mutations of bladder cancer were visualized by the OncoPrinter function of cBioPortal ( http://www.cbioportal.org/). Immune cell infiltration data for bladder cancer tissue samples were obtained from TCGA and calculated by the Cell-type Identification by Estimating Relative Subsets of RNA Transcripts (CIBERSORT) algorithm ^[ 21] ^.

### Study population

A case-control study was performed with 580 bladder cancer cases recruited from Nanjing in May 2003 and 1101 cancer-free controls randomly selected for matched age (± 5 years) and sex to the cases in the same geographic area during the same time period. The demographic characteristics of all participants were reported in previous studies, and the current study was approved by the Institutional Review Board of Nanjing Medical University with an informed consent form each participant ^[ 6, 22– 23] ^.

### Selection of apaQTL-genes and apaQTL-SNPs

We performed differential expression analysis for differences between bladder cancer tissues and adjacent normal tissues from the TCGA database for apaQTL-genes with quality control conditions of call rate 95% and *P* < 0.05.

**Figure 1:**
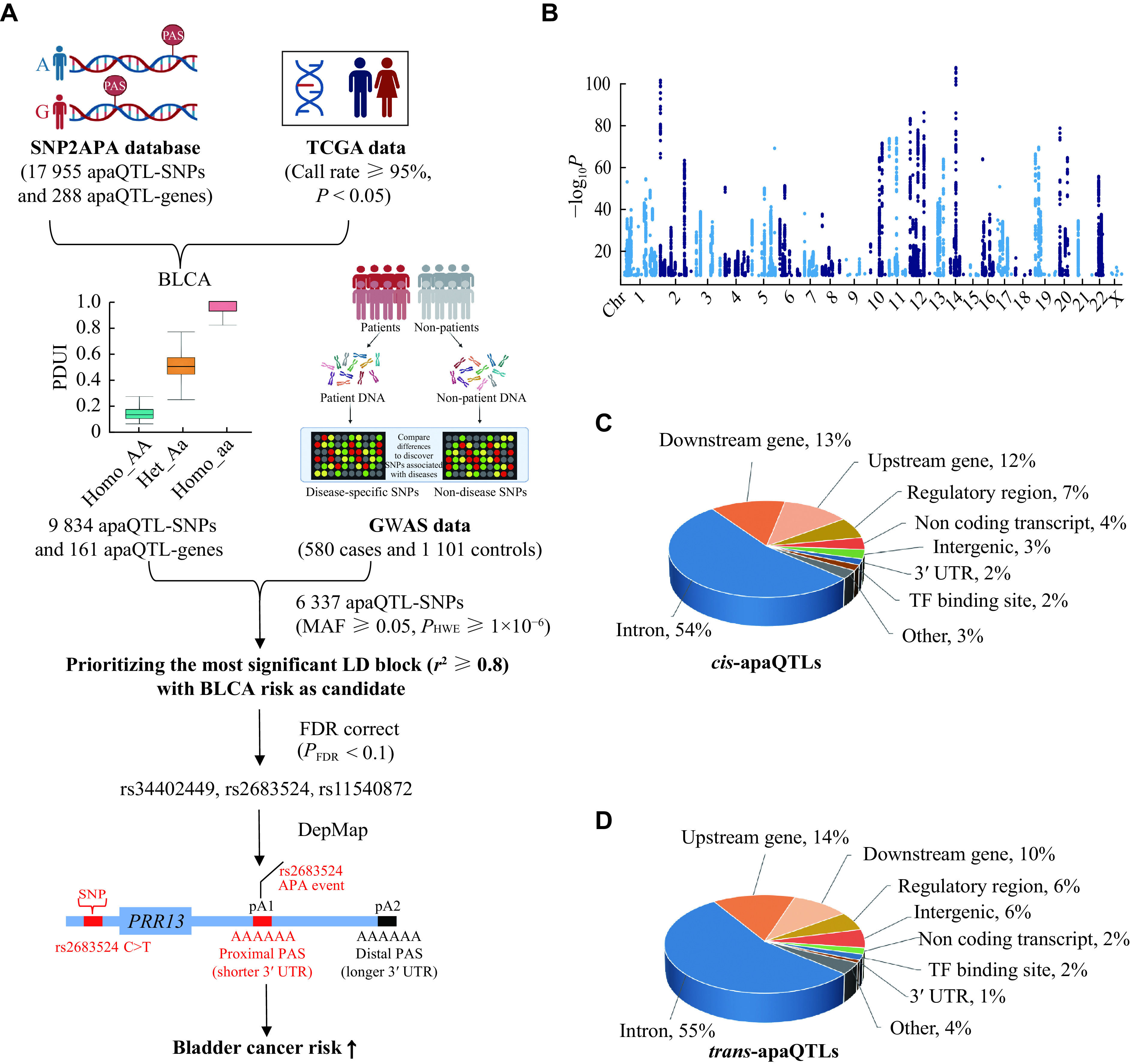
Identification and characterization of apaQTL-SNPs in bladder cancer.

After initial gene selection, we carried out systematic quality control of apaQTL-SNPs with the following exclusion criteria: (1) genotyping call rate < 95%, (2) minor allele frequency (MAF) < 0.05, and (3) *P-*value of Hardy-Weinberg equilibrium (HWE) < 1 × 10 ^−6^. Next, we conducted the screening of apaQTL-SNPs using linkage disequilibrium (LD) analysis ( *r* ^2^ ≥ 0.8). The flowchart of the SNP screening process is shown in ***Fig. 1A***.

### Gene expression analysis

Using the DepMap database, we obtained gene effect scores for bladder cancer cell lines from a CRISPR knockout screen released by Broad's Achilles and Sanger's SCORE projects ^[ 24]^ to select essential genes. The Cancer Cell Line Encyclopedia (CCLE) data in the DepMap database showed the expression levels of apaQTL-genes in several cell lines, and the expression in multiple tumor tissues and adjacent normal tissues by TIMER 2.0 ( http://timer.cistrome.org/) and Human Protein Atlas database (HPA, http://www.proteinatlas.org/). Additionally, we downloaded mRNA expression data of the apaQTL-genes in bladder cancer tissues from the TCGA database ( https://portal.gdc.cancer.gov/) and Gene Expression Omnibus database (GEO, https://www.ncbi.nlm.nih.gov/gds/, GSE38264) ^[ 25] ^. According to the TISIDB website ( http://cis.hku.hk/TISIDB/), we observed an association between the apaQTL-genes and immune subtypes of bladder cancer.

### Functional annotation for SNPs

RNAfold ( http://rna.tbi.univie.ac.at//cgi-bin/RNAWebSuite/RNAfold.cgi) predicted RNA secondary structure by calculating the minimum free energy (MFE). The RegulomeDB ( https://regulomedb.org/) database provided chromatin states and summary scores. 3DSNP ( https://omic.tech/3dsnpv2/) predicted enhancer and promoter. HaploReg4.1 ( http://pubs.broadinstitute.org/mammals/haploreg/haploreg_v4.1.php) showed peak DNase sensitivity and selected eQTL.

APAatlas ( https://hanlab.uth.edu/apa/) is a database for exploring the APA landscape across tissues and investigating the use of APA. The SNP2APA database quantified dynamic APA events by PDUI values, the boxplots represented the alternative polyadenylation levels in individuals carrying different genotypes ^[ 20] ^. The 3′aQTL database ( https://wlcb.oit.uci.edu/3aQTLatlas/) was used to recognize common genetic variants linked to differences in the 3′ UTR use. The eQTLGen consortium ( https://www.eqtlgen.org/cis-eqtls.html) performed *cis*- and *trans*-eQTL analyses using a blood-derived expression from 31684 individuals. PolyA_DB ( https://exon.apps.wistar.org/PolyA_DB/v3/index.php) and PolyASite 2.0 ( https://polyasite.unibas.ch/) were used to predict the polyA sites of genes.

### Statistical analysis

The feature distribution was calculated for comparisons between bladder cancer cases and controls by Chi-squared tests and Student's *t*-tests. The Chi-squared test of goodness-of-fit was applied to HWE of SNPs, derived from the allele frequencies of the control group. Associations between genetic variants and bladder cancer risk were assessed by calculating odds ratios (ORs) and corresponding 95% confidence intervals (CIs) using logistic regression models, with adjustment for age, sex, and smoking status. We used the false discovery rate (FDR) to correct *P*-values for the false positive rate generated after multiple comparisons. Stratified analyses were conducted by demographics. We assessed the joint effects of the associated apaQTL-SNPs on bladder cancer by counting the number of risk genotypes and risk factors. *P*-value < 0.05 and *P*_FDR_ < 0.1 were considered statistically significant. Finally, we also performed the false-positive report probability (FPRP) to verify significant results from the combined subjects ^[ 26] ^. All statistical analyses were conducted by R 4.1.3 and PLINK 1.09.

## Result

### Identification and characterization of apaQTL-SNPs in bladder cancer

As shown in ***Fig. 1A***, in the SNP2APA database, a total of 17955 apaQTL-SNPs for bladder cancer were included, containing 17072 *cis*-apaQTLs and 883 *trans*-apaQTLs ( ***Fig. 1B***). After removing duplicates, we identified 16470 SNPs in *cis*-apaQTLs and 819 SNPs in *trans*-apaQTLs. To characterize the genomic distribution of these apaQTL-SNPs, we conducted functional annotation and classification for SNPs of *cis*-apaQTLs and *trans*-apaQTLs using the Variant Effect Predictor. The results showed that the SNPs of *cis*-apaQTLs were enriched in the following regional categories: intron, downstream gene, upstream gene, regulatory region and non coding transcript; and the SNPs of *trans*-apaQTLs were enriched in the following regional categories: intron, upstream gene, downstream gene, regulatory region and intergenic ( ***Fig. 1C*** and ***1D***).

### Genes affected by apaQTL-SNPs play crucial roles in cancer development

**Figure 2:**
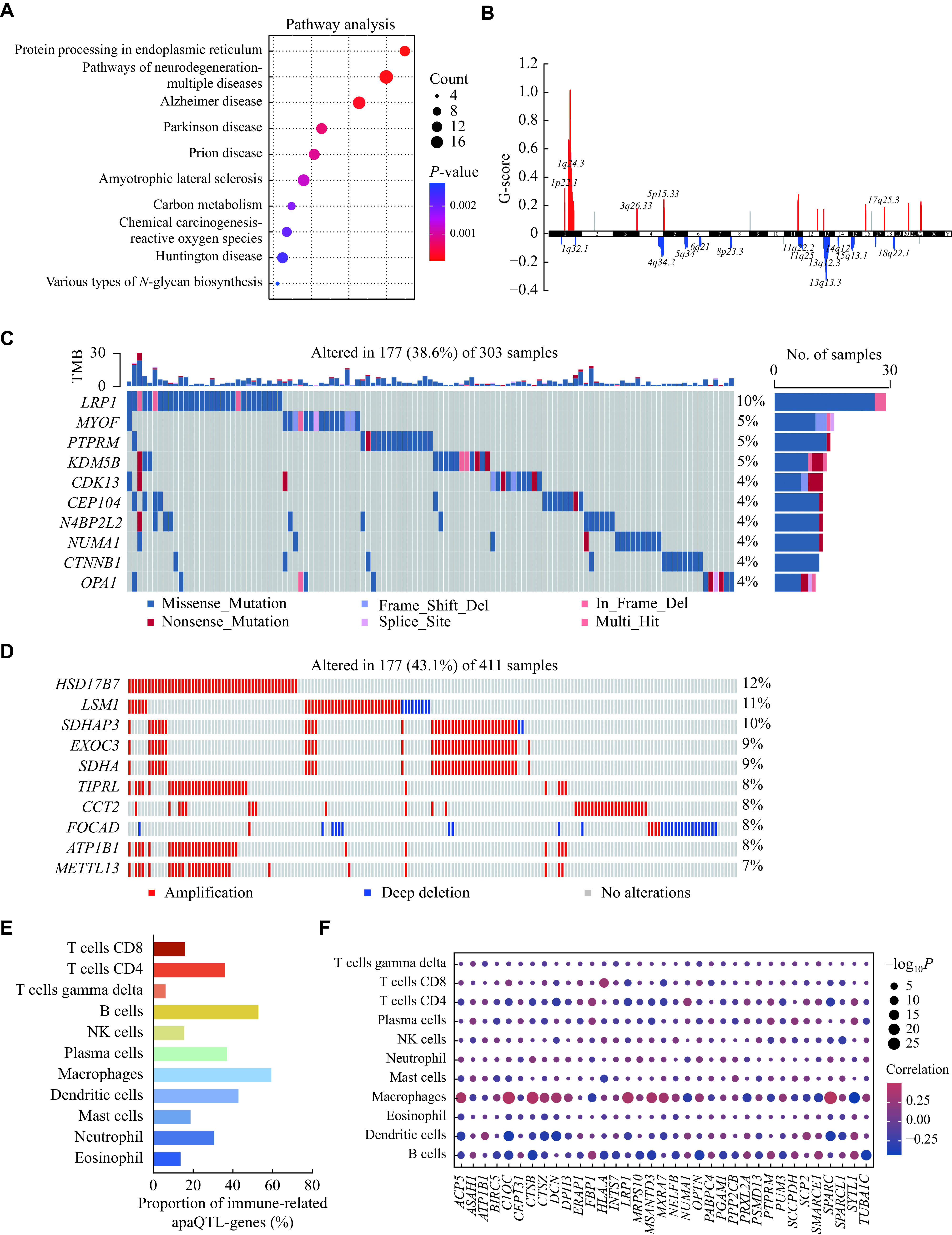
Genes affected by apaQTL-SNPs play crucial roles in cancer development.

In the SNP2APA database, a total of 288 apaQTL-genes were affected by apaQTL-SNPs ( ***Fig. 1A*** and ***Supplementary Table 1*** [available online]). To illustrate the potential contribution of apaQTL-genes to the development and treatment response of cancer, we used the KEGG analysis to determine whether apaQTL-genes predominantly existed in cancer hallmark pathways, and the results showed that multiple signaling pathways, such as protein processing in the endoplasmic reticulum and pathways of neurodegeneration-multiple diseases, were significantly altered ( ***Fig. 2A*** and ***Supplementary Table 2*** [available online]).

Recent studies have demonstrated that genes in SCNAs may serve as disease critical oncogenic drivers ^[ 27] ^. Thus, after analyzing the SCNAs data of TCGA bladder cancer tissues, we found that 17 chromosomal regions had massive amplifications and deletions ( ***Fig. 2B***). Furthermore, we assessed the somatic mutation frequency of the apaQTL-genes in bladder cancer. The results showed that *LRP1* had the highest mutation frequency (10%) ( ***Fig. 2C***). The top 10 copy number alterations of the apaQTL-genes are shown in ***Fig. 2D***, and *HSD17B7* had the highest amplification. The above results suggest that amplifications or deletions of the apaQTL-genes may provide the possibility of potential predictive markers for the treatment response of diseases and enlarge the understanding of clinically targeted chemotherapy.

Given that immune cells, along with their characteristic cytokines, were involved in cancer progression ^[ 28] ^, we further evaluated the correlation between apaQTL-genes expression and immune cell infiltration, and found that most apaQTL-genes and immune cells, particularly macrophages and B cells, were significantly associated with high immune cell infiltration ( ***Fig. 2E*** and ***2F***). The significant correlations between the apaQTL-genes and immune cell infiltration responses suggested that these genes could regulate immune cell infiltrating extent, affecting the development and progression of cancer to different degrees. For example, C1QC, one of the components of complement C1, was shown in a study to have its expression positively correlated with some immune cells, such as macrophages ^[ 29] ^, which is consistent with the results of our analysis ( ***Fig. 2F***). Therefore, in-depth analysis of the cross effect of apaQTL-genes and the immune system may provide potential strategies for a better prevention, treatment, and prognosis for bladder cancer.

### Associations of the apaQTL-SNPs with bladder cancer risk

**Figure 3:**
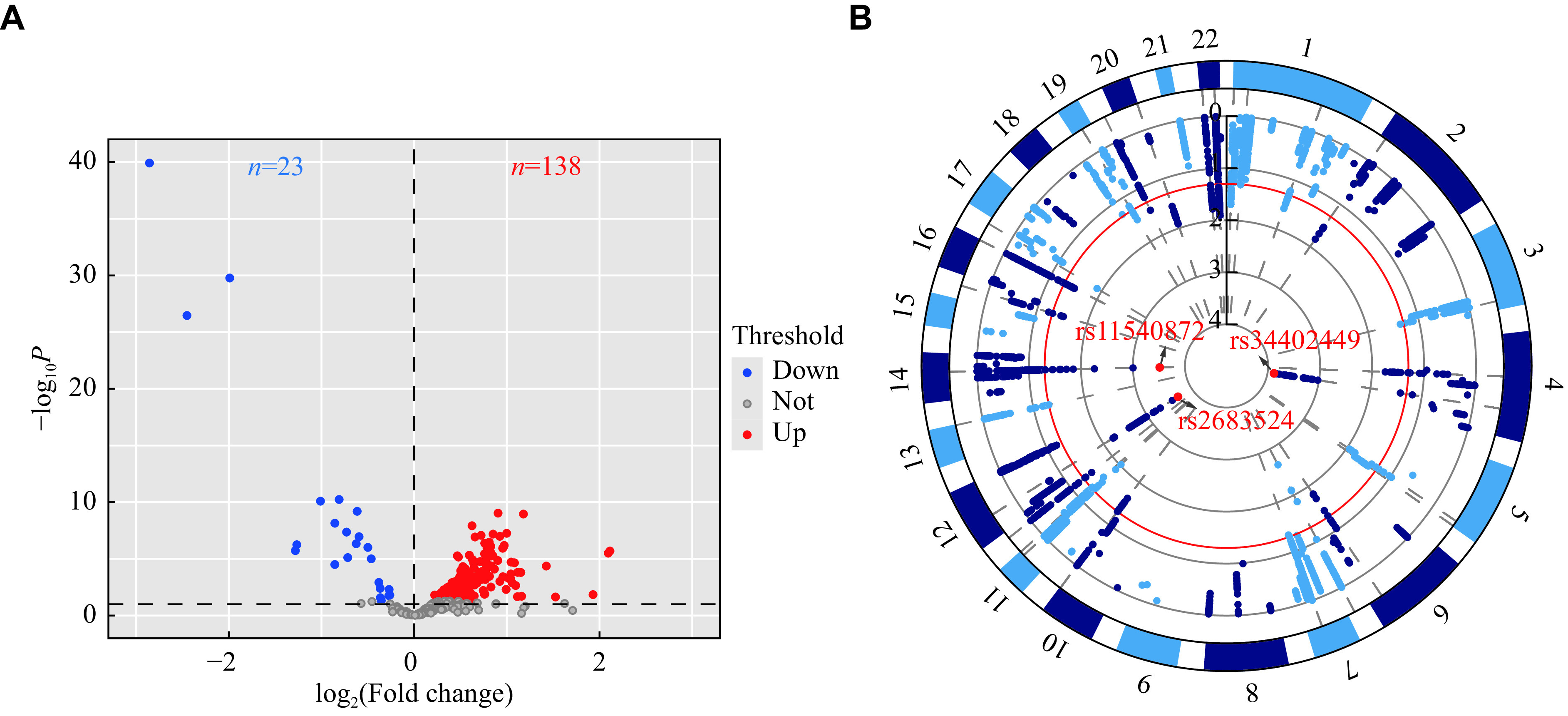
SNP selection and association with bladder cancer risk.

Although we had some understanding of apaQTL-genes through systematic characterization, we could not fully elucidate the associations between genetic variants and the risk of bladder cancer. To identify genetic variants that may influence bladder cancer risk by potential APA events, we integrated the apaQTL-SNPs and GWAS data for bladder cancer (580 cases and 1101 controls with the characteristics as previously described) ^[ 6, 22- 23] ^. First, we performed differential expression analysis of 288 apaQTL-genes using the TCGA bladder cancer database of 161 apaQTL-genes ( ***Supplementary Table 3 ***, available online) with 9 834 apaQTL-SNPs ( ***Supplementary Fig. 1A ***, available online). Of these genes, 138 genes were highly expressed and 23 genes were expressed at a low level in bladder cancer tissues ( ***Fig. 3A***). After quality control, we extracted 6 637 apaQTL-SNPs for further analyses ( ***Fig. 3B*** and ***Supplementary Fig. 1B ***). Through LD analysis and FDR correction, only three SNPs (rs34402449 in *ZNF330*, rs2683524 in *PRR13*, and rs11540872 in *CHURC1*) remained ( *P*_FDR_ < 0.1) to be significantly associated with an increased risk of bladder cancer ( ***Table 1***). These analyses were also repeated for the European population of 5930 bladder cancer cases and 5468 controls from dbGAP ( ***Supplementary Table 4 ***, available online), but none of the three SNPs were susceptible variants in the European population.

**Table 1:** Association between three significant apaQTL-SNPs and bladder cancer risk

SNPs	Chr	Gene	Position	*β* ^a^	*P* ^b^	Allele ^c^	MAF		*P*_HWE_	Adjusted OR (95% CI) ^d^	*P*	*P*_FDR_
							Cases	Ctrl				
rs34402449	4	*ZNF330*	142130931	−0.28	7.59×10 ^−12^	C/A	0.385	0.321	0.725	1.36 (1.16–1.58)	1.33×10 ^−4^	9.68×10 ^−2^
rs2683524	12	*PRR13*	53814853	−0.26	5.38×10 ^−11^	C/T	0.256	0.198	0.341	1.38 (1.16–1.63)	2.03×10 ^−4^	9.68×10 ^−2^
rs11540872	14	*CHURC1*	65414738	0.79	1.12×10 ^−43^	C/G	0.162	0.116	0.553	1.47 (1.19–1.82)	3.06×10 ^−4^	9.68×10 ^−2^
^a^Effect size of SNP on PDUI, calculated by linear regression using a computationally efficient eQTL analysis called Matrix eQTL. ^b^ *P*-value of apaQTL, calculated by Matrix eQTL. ^c^Reference/effect allele. ^d^Adjusted for age, sex and smoking status in the logistic regression model. Abbreviations：SNPs, single nucleotide polymorphisms; Chr, chromosome; MAF, minor allele frequency; Ctrl, controls; HWE, Hardy-Weinberg equilibrium; OR, odds ratio; CI, confidence interval; FDR, false discovery rate.												

Going a step further, we used multivariable logistic regressions with adjustment for age, sex, and smoking status to evaluate the associations of these three SNPs with bladder cancer risk ( ***Supplementary Table 5 ***, available online). Following a dominant genetic model, the *ZNF330* rs34402449 CA/AA, *PRR13* rs2683524 CT/TT, and *CHURC1* rs11540872 CG/GG were all associated with an increased risk of bladder cancer (adjusted OR = 1.447, 95% CI: 1.169–1.791 for rs34402449; adjusted OR = 1.464, 95% CI: 1.190–1.801 for rs2683524; adjusted OR = 1.497, 95% CI: 1.188–1.887 for rs11540872). Moreover, in a recessive genetic model, individuals carrying the rs2683524 TT genotype, compared with those with CC/CT genotypes, had a 57.2% increased risk of bladder cancer (adjusted OR = 1.572, 95% CI: 1.014–2.439). The rs11540872 GG genotype also notably increased bladder cancer risk, compared with the CC genotype, with a 2.367-fold risk elevation (adjusted OR = 2.367, 95% CI: 1.079–5.196). In addition, the associations of these three SNPs as estimated in multivariabel models remained insignificant in European populations ( ***Supplementary Table 6 ***, available online).

### Stratification analysis and joint effects of apaQTL-SNPs and bladder cancer risk

In stratified analyses by age, sex, and smoking status subgroups, we assessed the associations between the three candidate apaQTL-SNPs and bladder cancer risk in Chinese populations ( ***Supplementary Fig. 2 ***, available online). The results showed that the CA/AA genotype of rs34402449 and the CT/TT genotype of rs2683524 were associated with significantly increased bladder cancer risk in the elderly subgroup (age > 65), compared with those with the CC genotype for both rs34402449 and rs2683524 (adjusted OR = 1.503, 95% CI: 1.212–1.864 for rs34402449; adjusted OR = 1.505, 95% CI: 1.196–1.894 for rs2683524), respectively. In addition, we found that the male subgroup had a markedly increased bladder cancer risk in individuals with the T allele of rs2683524 and the G allele of rs11540872 than those with the C allele (adjusted OR = 1.371, 95% CI: 1.141–1.647 for rs2683524; adjusted OR = 1.511, 95% CI: 1.197–1.908 for rs11540872), respectively. In the never-smoking subgroup, we observed a significantly increased risk of bladder cancer associated with the CA/AA genotype in rs34402449 in comparison with the CC genotype (adjusted OR = 1.478, 95% CI: 1.210–1.807). However, the European population still had no significant results in these analyses ( ***Supplementary Fig. 3 ***, available online).

Additionally, to determine whether the apaQTL-associated SNPs and smoking status were independent or not, we examined the joint effects of the three apaQTL-SNPs and smoking status on the risk of bladder cancer ( ***Supplementary Table 7 ***, available online). We included the unfavorable genotypes ( *ZNF330* rs34402449 CA/AA, *PRR13* rs2683524 CT/TT, and *CHURC1* rs11540872 CG/GG) as risk genotypes as the number of risk genotypes (NRGs) into a genetic score. The results showed that compared with the individuals without risk genotype, those with several risk genotypes had a higher bladder cancer risk, and those with an increase of quantity risk genotype also had an increased risk ( *P*_trend_ = 7.18 × 10 ^−9^). When taking smoking into consideration, compared with those not carrying risk genotypes/factors, those having four risk genotypes/factors had a 6.542-fold increased risk for the development of bladder cancer ( *P* = 1.91 × 10 ^−5^, *P*_trend_ = 2.87 × 10 ^−12^). Similarly, we performed a stratified analysis for the association between NRGs and bladder cancer risk. There was a significant difference in the male group, compared with the female group (adjusted OR = 1.720, 95% CI: 1.296–2.303, ***Supplementary Table 8 ***, available online). However, there was no heterogeneity between the covariables and these three apaQTL-SNPs or NRGs.

### FPRP results

The FPRP was applied to reduce the probability of false-positive findings ^[ 30] ^. FPRP noteworthiness value was set at 0.2 and the prior probabilities were set according to their functions. As shown in ***Supplementary Table 9*** (available online), at the prior probability of 0.1, the significant findings for the apaQTL-SNP rs34402449 C>A, rs2683524 C>T, and rs11540872 C>G polymorphism remained noteworthy, except for the results on rs26835224 C>T for those with risk factors and the recession model, indicating that our findings remain noteworthy.

### Expression levels of *PRR13* in tumor tissues and cell lines

**Figure 4:**
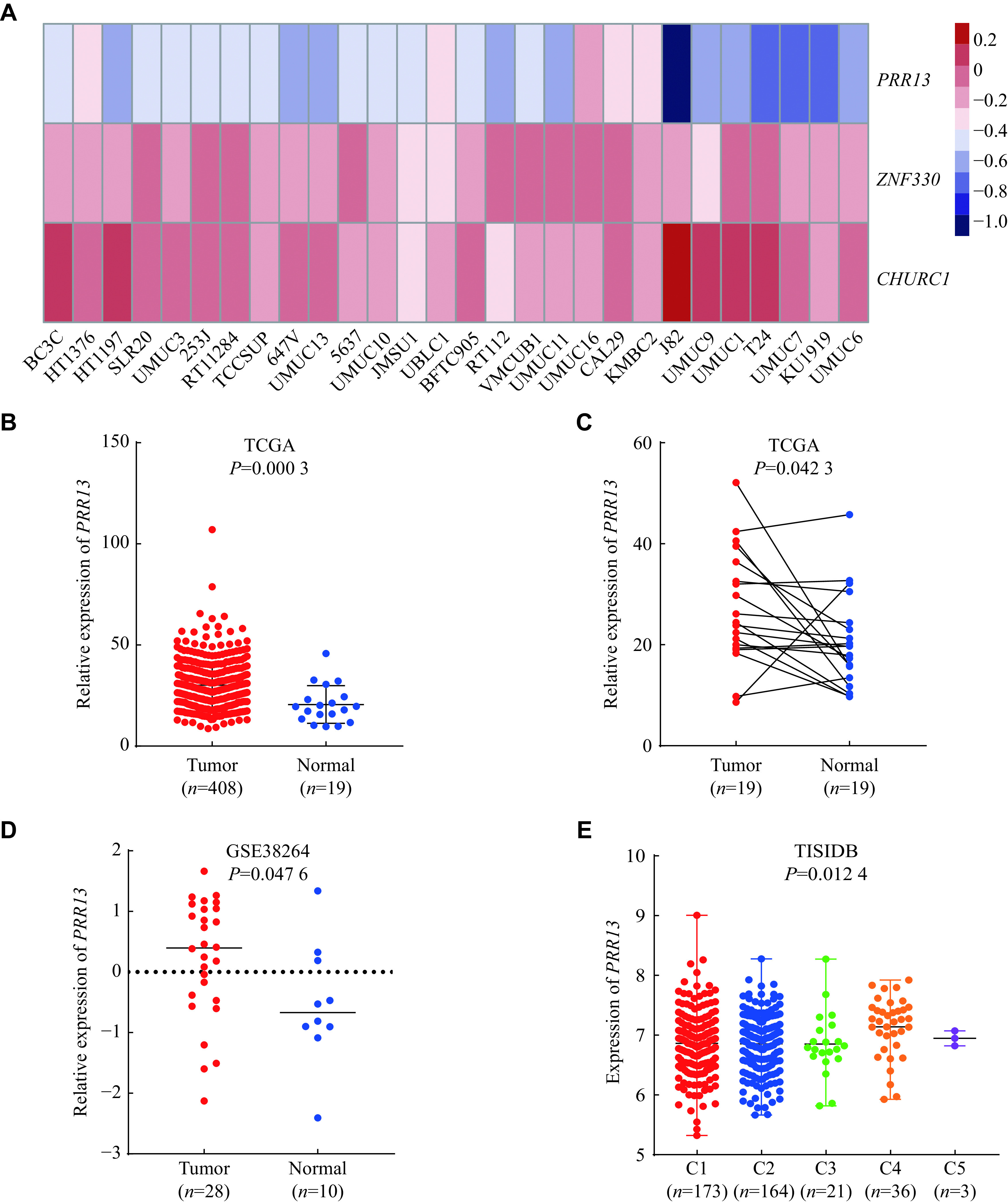
Expression level of *PRR13* in bladder cancer tissues and normal tissues.

To explore the function of apaQTL-genes in bladder cancer, we obtained gene effect scores of these three apaQTL-genes using a CRISPR knockout screen from the DepMap database. The results showed that *PRR13* had the minimum negative score, compared with *ZNF330* and *CHURC1* in 28 bladder cancer cell lines, suggesting that knockout of *PRR13* had the most significant inhibitory effect on the viability of bladder cancer cells ( ***Fig. 4A***). *PRR13* was further included in the follow-up study. The CCLE data allowed for the observation of *PRR13* expression in multiple cell lines ( ***Supplementary Fig. 4A ***, available online). *PRR13* was aberrantly expressed in multiple tumor tissues and adjacent normal tissues ( ***Supplementary Fig. 4B ***– ***4E ***). We evaluated the expression of *PRR13* in bladder cancer using the TCGA database. The results showed that *PRR13* expression in bladder cancer tissues was remarkably increased relative to adjacent normal tissues ( *P* = 0.0003 in unpaired samples, ***Fig. 4B***; *P* = 0.0423 in paired samples, ***Fig. 4C***). We obtained similar results in the GSE38264 dataset of the GEO database ( *P* = 0.0476; ***Fig. 4D***). In cluster analysis, the immune expression signatures of non-hematologic tumors in TCGA were clustered into six immune subtypes, which were used to characterize intratumoral immune states ^[ 24] ^. According to the TISIDB website, we evaluated the expression distribution of *PRR13* in the immune subtypes of bladder cancer, and found that its expression varied among different immune subtypes, and that the highest expression of C4 was observed (lymphocyte depleted) ( ***Fig. 4E***).

### SNP rs2683524 regulates *PRR13* expression *via* alternative polyadenylation

**Figure 5:**
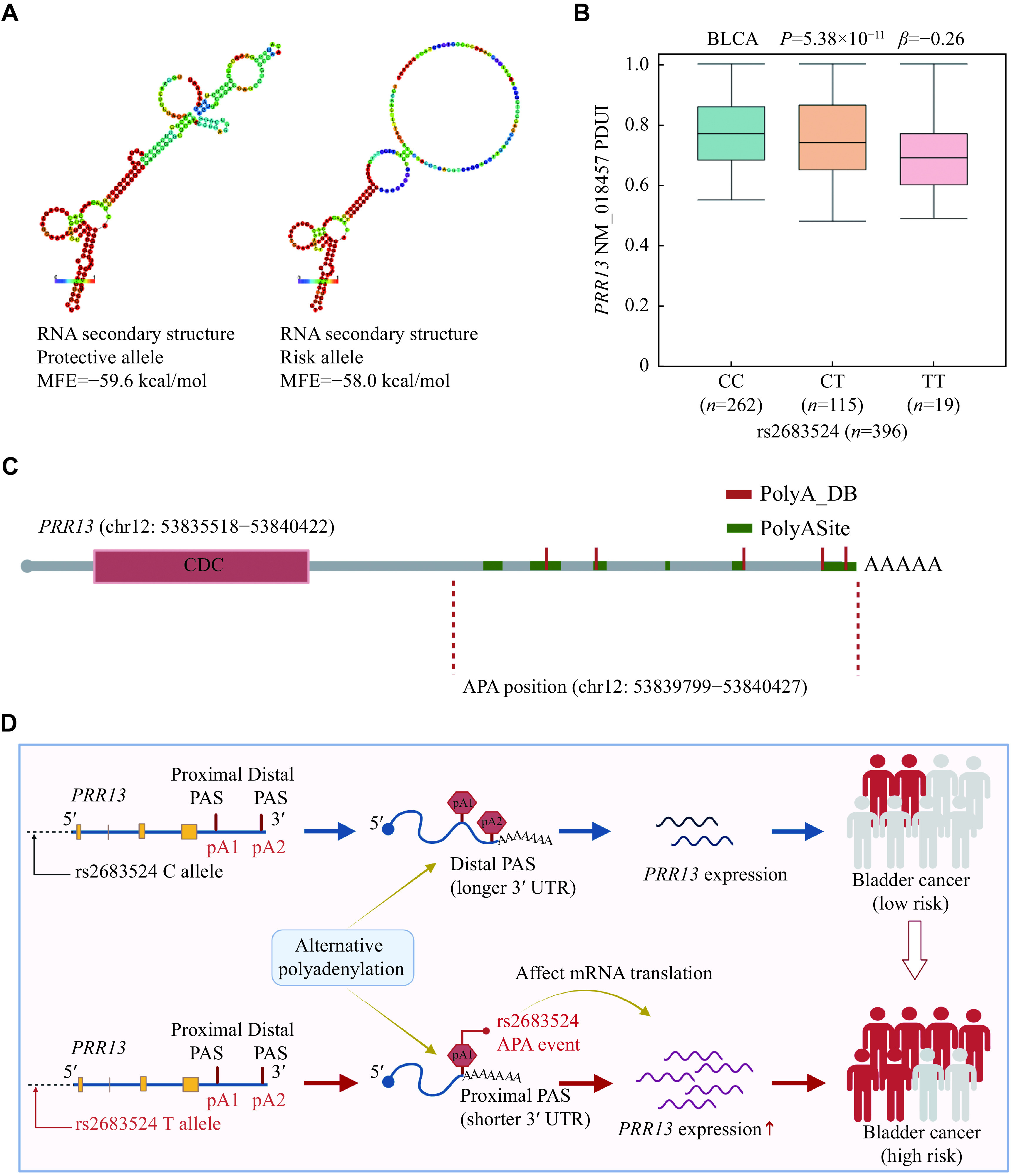
The role of rs2683524 in APA and *PRR13* expression.

The functional annotation of rs2683524 was performed using RegulomeDB, 3DSNP, HaploReg4.1, and RNAfold ( ***Supplementary Table 10 ***, available online), in which 3DSNP showed that rs2683524 was in the enhancer state in 25 cell types and the promoter state in four cell types. RNAfold predicted that rs2683524 C>T caused changes in MFE (ΔMFE = 1.6 kcal/mol), and the secondary structure of RNA for protective and risk alleles is shown in ***Fig. 5A***.

The APAatlas database showed an APA landscape of *PRR13* in different tissues ( ***Supplementary Fig. 5A*** and ***5B ***, available online), indicating that alternative polyadenylation of *PRR13* was prevalent in all types of tissues. The PDUI value was an indicator to quantify dynamic APA events, and SNP rs2683524 C>T was associated with the reduced PDUI values, suggesting that the proximal polyA sites were preferentially selected to form a shorter 3′ UTR, which was highly associated with APA events of *PRR13* in bladder cancer ( *P* = 5.38 × 10 ^−11^; *β* = −0.26, ***Fig. 5B***). In addition, we also observed similar results for rs2683524 highly-linked SNPs (rs11170549 and rs11170550) in whole blood in the 3′aQTL database ( ***Supplementary Fig. 5C*** and ***5D***). Meanwhile, the *cis*-eQTL effect from the eQTLGen consortium showed that *PRR13* rs2683524 C>T had a significantly upregulated *cis*-QTL effect ( *P* = 3.92 × 10 ^−77^, Z-core = 18.5895, ***Supplementary Table 11 ***, available online). Combined with the above-presented results, the apaQTL analysis in bladder cancer showed that the T allele of SNP rs2683524 preferentially selected the proximal PAS of *PRR13*, forming the shorter 3′ UTR, compared with the C allele, and the eQTL analysis showed that rs2683524 C>T could significantly increase the expression levels of *PRR13*.

Based on the APA positions affected by SNP rs2683524, we predicted the polyA sites where SNP rs2683524 might affect *PRR13* using two websites. PolyA_DB database predicted five possible polyA sites for *PRR13*, and the PolyASite database predicted six intervals of potential polyA sites ( ***Supplementary Tables 12*** and ***13 ***, available online). There was an overlap between the predictions of the two databases with some confidence ( ***Fig. 5C***). This provides the basis for subsequent studies, and more experiments are needed to validate our findings in the future.

## Discussion

In the current study, we first comprehensively characterized apaQTL-SNPs and apaQTL-genes by function classification, gene function, copy number variation level, and correlation with immune infiltration. Then, the associations between apaQTL-SNPs and risk of bladder cancer were assessed in a case-control study. We further selected *PRR13* based on cell viability prediction and found that it was abnormally upregulated in bladder cancer tissues. Moreover, we found that the T allele of SNP rs2683524 preferentially selected the proximal polyA site of *PRR13* to form the shorter 3′ UTR than the C allele, resulting in the higher translation efficiency and expression level of *PRR13*, contributing to the development of bladder cancer ( ***Fig. 5D***). As far as we know, this is the first study that has evalutated the associations between alternative polyadenylation related genetic variants and bladder cancer risk.

In clinical practice, therapeutic agents to suppress tumors can be designed by identifying molecular targets known to have oncogenic effects ^[ 31] ^. With this in mind, we analyzed apaQTL-genes in cancer signaling pathways, somatic mutations and immune infiltration. The KEGG analysis revealed significant changes in several signaling pathways. Analysis of the copy number variation level showed that apaQTL-genes had significant amplifications and deletions. In addition, the correlation analysis showed that apaQTL-genes were significantly associated with immune cell infiltration. Overall, the apaQTL-genes provide some clues for clinical treatment application and can further be used as a potential predictive marker for disease treatment response.

Proline-rich protein 13 ( *PRR13*, synonyms: *TXR1*) is located in the 12q13.13 region that encoded an 18-kDa proline- and serine-rich nuclear protein, and is closely correlated with the paclitaxel resistance ^[ 32] ^. Studies have shown that *PRR13* is highly expressed in tumor tissues, and thrombospondin 1 ( *THBS1*, synonyms: *TSP1*) may induce apoptosis *via* CD47 following taxane-induced damage to the cellular microtube network ^[ 33– 34] ^; up-regulation of *PRR13* confers resistance to taxanes by inhibiting *TSP1* ^[ 34– 35] ^. One study showed that overexpression of *PRR13* was significantly associated with downregulated expression of *TSP1*, and that patients with favorable genotypes (high *TXR1*/ *TSP1* expression) had significantly higher disease control rates, median time to tumor progression and median overall survival, compared with patients with unfavorable genotypes (low *TXR1*/ *TSP1* expression) ^[ 35] ^.

SNP rs2683524, located approximately 20 kb upstream of *PRR13*, was remarkably correlated with bladder cancer risk. A series of analyses showed that the CT/TT genotypes of SNP rs2683524 remarkably increased bladder cancer risk in male and older individuals than in femal and younger individuals, respectively, suggesting that rs2683524 may serve as a biomarker for bladder cancer susceptibility in male and older individuals. To investigate the results in other populations, we performed the same analyses in European populations and found that it was insignificant, possibly due to ethnic differences in genetic backgrond and lifestyles. In addition, smoking was considered the main and most modifiable risk of bladder cancer ^[ 30] ^. The joint effect results showed that the individual bladder cancer risk increased with the increase of the number of risk genotypes. After accounting for smoking as a risk factor, individuals with all four risk genotypes/factors had a 6.542-fold increased risk of bladder cancer, compared with those without. We have also shown that smoking as a risk factor can have a combined effect with genetic factors, suggesting that changing smoking behaviour may prevent the development of bladder cancer.

APA events of *PRR13* are ubiquitous in various tissues. To quantify APA events, PDUI values were an innovative and intuitive ratio ^[ 21] ^. Using the TCGA database, we found that SNP rs2683524 had a significant apaQTL effect on *PRR13* in bladder cancer. With rs2683524 C>T, PDUI values decreased, indicating a preference for proximal polyA sites. However, the TCGA database is mainly derived from European and American populations, and thus it is necessary to expand the population samples to verify the ethnic differences of rs2683524. Meanwhile, there was a significant up-regulated *cis*-eQTL effect of rs2683524 C>T that regulated the expression of *PRR13*. Therefore, we conclude that the SNP rs2683524 C>T may cause *PRR13* to preferentially select the proximal polyA site to form a shorter 3′ UTR, which may improve the translation efficiency and elevate the expression of *PRR13*, thus contributing to the development of bladder cancer.

Collectively, our results highlight a link between APA-related genetic variant rs2683524 and bladder cancer risk. We have shown that SNP rs2683524 can affect the APA events of *PRR13*, which in turn affects gene expression. In addition, we tentatively predict that SNP rs2683524 may affect the polyA sites of *PRR13*, providing the basis for future studies. These results provide new insights into the role of APA in bladder cancer risk and may help identify possible biomarkers for bladder cancer susceptibility.
